# Scaling up coral spawn collection: Impacts of method and timing on *Acropora valida* larval quality

**DOI:** 10.1371/journal.pone.0331461

**Published:** 2025-09-11

**Authors:** Christina Langley, Christopher Doropoulos, Dexter dela Cruz, Peter L. Harrison

**Affiliations:** 1 Faculty of Science and Engineering, Southern Cross University, East Lismore, New South Wales, Australia; 2 CSIRO Environment, St Lucia, Queensland, Australia; University of the Ryukyus, JAPAN

## Abstract

Scaling up coral reef restoration to ecologically relevant scales presents a significant challenge during propagule collection. Mass coral spawning events are a vast source of propagules for reef restoration, but these events are typically limited to a few nights annually. Various methods of spawn collection following spawning events are available, ranging from traditional small-scale collection to industrial large-scale collection. However, comparisons between methods and potential effects on larval integrity are poorly understood. In this laboratory-based study, different methods of spawn collection – buckets, nets, and diaphragm pumping – were tested at various time points following spawning to explore potential impacts on embryo integrity, larval size, rate of deformities, and larval settlement. Results indicated that the collection method and, especially, the timing of collection, were critical. While bucket collection had minimal impact on embryo integrity, net and pump techniques caused high embryo fragmentation (>45%) at 5–11 hours post-fertilisation when embryos were >8 cells. This significantly reduced the average size of developing larvae in net and pump collections 3–11 hours post-fertilisation. When collections took place within the first hour of fertilisation before embryo cleavage, using any collection method resulted in minimal fragmentation (<4%). In general, net samples had larger larvae than pumped samples. However, larger larvae appeared to be more prone to deformities, and deformed larvae exhibited reduced settlement success (4% deformed vs 25% intact). These results highlight how large-scale spawn collections can be conducted without compromising larval quality when timed carefully, offering practical guidance for scaling coral reef restoration efforts.

## Introduction

Active coral reef restoration efforts and climate change mitigation are urgently required to reverse the global decline of coral reefs and future predictions of ecosystem collapse [1–3]. With the area of degraded reef systems expanding, large-scale coral reef restoration interventions are being sought. Scaling up coral reef restoration to ecologically relevant scales represents a major challenge. However, restoration methods using sexually reproduced coral larvae have the potential to meet the requirement of scalability [2,4,5].

Mass coral spawning events are a source of vast amounts of sexually produced coral larvae in the wild [6], which can be used to scale up reef restoration efforts with minimal impact to natural populations [7–11]. Mass coral spawning events on the Great Barrier Reef (GBR) typically occur over 2–3 main nights during two or three lunar cycles each year, in late spring and summer [6,12,13]. Most coral species are hermaphroditic broadcast spawners, and many of these release their gametes into the water column in positively buoyant bundles of eggs and sperm [5,6,14,15]. The spawned gamete bundles float to the sea surface before breaking apart and fertilising, making them ideal for targeted collection [15–17]. Under favourable conditions, mass spawning events can form dense slicks representing a highly concentrated source of coral gametes [18,19]. However, the gametes become more dispersed when coral densities are low or windy conditions occur during spawning [20,21], making them more challenging to collect. Additionally, the formation, movement, and persistence of spawn slicks are also strongly influenced by oceanographic processes such as currents, eddies, and surface turbulence, which can either concentrate gametes into predictable aggregations or disperse them over broad areas [18,22]. Therefore, scaling up the collection of coral gametes requires access to different methods best suited for different reef states, reef habitats and environmental conditions during spawning events.

Traditional smaller-scale collections of coral gametes ex situ have used active collection, skimming gametes gently from the surface using small collection containers held just below the surface of the water on the edge of the slick, creating suction [4,23,24], or in situ from the sea surface using buckets or nets [10,11,19,25], as well as passive collection methods such as placing mesh cones over individual gravid coral colonies [23,26,27]. Larger-scale in situ versions of passive collection methods include the coral larval cradle [28], which is a bundle collection system where large conical nets are placed over many colonies, and the bundles are fed into larval rearing pools via a connecting hose. Another passive collection system, not limited to the collection of bundles within a specific area, is the spawn catcher with an inflatable boom system [11] to passively and efficiently capture samples of coral spawn slicks [25,29]. The success of the spawn catcher systems depends on the quality of the predicted location of slick formation and environmental conditions [22], as the location of spawn catchers is fixed prior to spawning events. In most cases, searching for spawn slicks is necessary, requiring active spawn collection from moving vessels. Simple collection methods can be carried out using buckets for dense spawn slicks and modified net scoops for dispersed coral spawn [25,29]. More complex active spawn collection methods include the use of remote-controlled robots [30] and industrial-scale pumping of spawn directly from slicks or combined with oil booms to concentrate the coral spawn [7]. These collection methods result in different levels of shear stress to the coral spawn.

After corals spawn, their buoyant gamete bundles break apart, and fertilisation begins. This is followed by embryogenesis, where the cells divide through cleaving and become more complex until they reach a more robust, round-shaped embryo stage [15,23,31,32]. The embryos are particularly vulnerable to fragmentation during embryogenesis as they lack a protective cell wall. Several studies investigating fragmentation during embryogenesis found that the fragmented embryos are able to develop into planula larvae that become competent and settle successfully [33–35]. This asexual fragmentation of embryos following sexual reproduction, known as polyembryony [36], may occur naturally at sea after spawning periods that coincide with rough sea states. Fragmentation during the 2–16 cell stage is known to result in individual larvae with sizes corresponding to the cleavage stage of the embryo from which they were derived [33]. Polyembryony in octocorals shows similar trade-offs between the number and size of propagules to maximise the number of surviving recruits [35], supporting the traditional trade-off theory, which suggests that an increase in fitness of one life history trait causes a decrease in fitness of another [37,38]. However, the advantages or disadvantages of polyembryony are uncertain [39].

The trade-off between size and fitness [40] has implications for the survival of smaller larvae [39,41], their dispersal [42], and post-settlement survival [43,44]. The survival of smaller blastomeres (i.e., cells produced during cleaving) is lower than larger ones, and the survival of the individual blastomere depends on the part of the original embryo to which it belonged [39]. Larval development time to competency has also been linked to larval size, with smaller larvae having shorter lifespans and reaching competency sooner, which may result in higher rates of self-recruitment, decreasing dispersion and connectivity between reefs [42,45] due to reduced energy resources [46,47]. Smaller larvae produce smaller recruits, which may require extended periods to reach the size-escape threshold [48,49], with higher mortality rates occurring in smaller coral recruits compared with larger recruits [44,50,51]. Shortening the time to competency may not be very important in reef restoration projects that use sexually produced coral larvae and direct seeding methods. In contrast, a delay in reaching the size-escape threshold is critical as early post-settlement mortality represents a significant bottleneck in successful restoration efforts using sexually produced coral larvae [9]. In light of these potential adverse effects of fragmentation, it is not surprising that most coral larval rearing protocols highlight the importance of handling the embryos gently after fertilisation to avoid fragmentation of the embryos [4,23,33,52–54].

Therefore, understanding the impacts of different collection methods and their timing following spawning during the harvesting of coral slicks is critical for scaling up reef restoration efforts, particularly in regions of the Pacific and Indian Oceans where large-scale synchronous spawning events are known to occur and large-scale restoration using sexually produced larvae is being developed or implemented.

In this study, we aimed to determine how different spawn collection methods – buckets, nets, and pumps – and the timing of their use after spawning affect coral embryo integrity, larval development, and settlement. To isolate the effects of collection method and timing, the study was conducted under laboratory conditions using a single species (*Acropora valida*) as a model organism, allowing us to control for variability in spawn slick timing, fertilisation status, and composition.

## Materials and methods

### Coral spawning and gamete collection

To investigate the effects of different collection methods and timing on coral embryo and larval development, fertilised eggs of *Acropora valida* were generated under laboratory conditions and then subjected to one of three collection approaches at four defined time points post-fertilisation. This design ensured a consistent and comparable starting point across all treatments.

Prior to the predicted coral spawning period in October 2023, 15 gravid colonies were collected at Snapper Bay (−18.6604, 146.5096) and on the Eastern side of Fantome Island (−18.66463, 146.5243), central GBR. The colonies were transported back to Orpheus Island Research Station (OIRS), where they were held in the outdoor flow-through aquarium facility and monitored after dark for signs of spawning.

The colonies spawned at 21:30–21:50 on the 3^rd^ of November 2023 (six nights after the full moon). Once the colonies showed signs of setting, they were separated into individual tubs with seawater to prevent fertilisation from occurring before gamete collection. Gamete bundles were collected from five different genotypes by skimming bundles from the water surface with cups. The bundles were separated using mesh strainers, and the eggs were rinsed, keeping the gametes from each genotype separate and free of cross-contamination until sufficient quantities of eggs for the experiment had been collected.

Fertilisation was initiated an hour post-spawning by combining all the eggs with a mixture of sperm from all genotypes into a ~ 70 L tub. Ten minutes after sperm addition, and before cleavage occurred, approximately a quarter of the eggs were rinsed with clean seawater to remove excess sperm. These were used for the first experimental time point. The remaining eggs were left undisturbed for one hour before being rinsed, and had ~ 97% fertilisation success. Fertilisation success was determined three hours post-fertilisation, by counting the number of cleaved embryos and unfertilised eggs in replicate 15 mL samples (n = 3).

### Embryo collections

Three different collection methods were compared: (1) skimming the surface using a bucket (20 L capacity), (2) scooping using a 150 µm mesh net attached to a modified commercially available pool scoop [25,29], and (3) pumping using a fuel-powered diaphragm pump with a 100 mm intake on the lowest flow setting (16 m^3^ h^-1^) [19].

Three 1000 L oval tanks were set up to hold the mock embryo slicks – one tank per collection method. For the collection methods using the bucket or net, a sample of fertilised eggs was added directly to each oval tank using a 0.5 L beaker, where the collection was carried out. The water surfaces were skimmed with the bucket or collection net, and collected samples were gently transferred into 2 L jars containing a small amount of filtered seawater to avoid eggs coming in direct contact with the sample jar upon transfer. The density of eggs in the jars for culturing was kept low, and filtered water was added so all jars had 1.5 L of water and ~0.5 eggs mL^-1^.

The collection method using the diaphragm pump required a slightly more complex configuration. It involved a 450 L cylindrical holding tank with a tapered bottom and a central outlet fitted with a valve. A 100 mm hose connected the outlet to the pump intake. Another 100 mm hose from the pump outlet was directed into the oval tank and submerged so that the eggs did not collide with the sides or bottom of the oval tank. Before depositing a sample in the holding tank, all lines were purged and primed, and the holding tank was filled with approximately 100 L of filtered seawater. Once the sample was received in the holding tank, the diaphragm pump was started, creating suction and a whirlpool formation in the holding tank, pushing water through the pump system and delivering the eggs into the oval tank. The eggs were then collected by scooping using sample jars.

All three collection methods were carried out simultaneously. Five replicate sample jars were collected for each method at each time point. Remaining embryos were strained out and discarded between each replicate sample, and oval tanks were drained and refilled between each time point. The first collection time point (t_0_) commenced 10 minutes after combining coral gametes and was completed within an hour after fertilisation, before cleavage occurred (t_0 _= one-hour post-fertilisation). The following collection time points were carried out at three- (t_1_), five- (t_2_), and 11-hours (t_3_) post-fertilisation.

**Fig 1:**
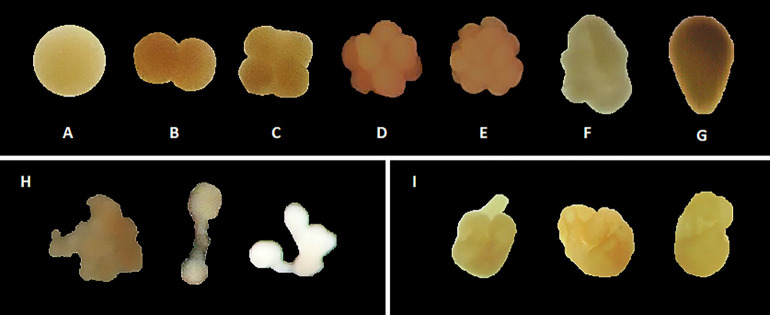
Embryo development stages of *Acropora* sp. used to classify embryos in fixed samples. A) 1-cell stage, B) 2-cell stage, C) 4-cell stage, D) 8-cell stage, E) >8-cell stage (morula), F) prawn chip stage, G) planula larval stage, H) examples of fragments and deformed embryos, and I) 3-day-old *A. valida* larvae with deformities.

Before (controls) and after each collection replicate (bucket, net, pump), samples were collected in 20 mL scintillation vials and fixed with a 10% buffered formalin solution in filtered seawater containing 20 g L^-1^ sodium ß-glycerophosphate. The fixative was pipetted carefully into the bottom of the vials, avoiding the floating eggs and embryos. Samples were assessed following collection trials to determine the immediate effect of the collection method and time on embryo integrity. The embryos were categorised by identifying the number of cells (i.e., two-cell stage, four-cell stage, etc.) and development stages of embryogenesis for *Acropora* sp. (Fig 1A-G). Embryos that fragmented into smaller counterparts were categorised by the number of cells, while embryos that did not conform to the usual categories and were highly irregular were classified as fragments and deformed embryos (Fig 1H).

### Larval culturing

After each collection time point, the 2 L sample jars with embryos were moved to a temperature-controlled room (~26.5°C) with continuous logging of temperature (miniDOT Clear Logger, © 2021 Precision Measurement Engineering, Inc.) under a 12:12 hour light:dark cycle with gradual ramping using artificial light (3.0 Plant Spectrum Bluetooth LED 32 W [lumens: 2.350 lm, colour temperature: 6500 K], Fluval®). Light intensity was programmed via the FluvalSmart app to simulate natural sunrise and sunset, with an approximate one-hour ramp up in the morning and a one-hour ramp down in the evening. Sample jars were left undisturbed for 24-hours before water was changed using 1 µm UV-filtered seawater (salinity 35‰, pH 8.4) and adding gentle aeration to the individual jars (n = 60). Due to logistical constraints, 50% water changes were carried out every other day.

### Larval size and health

Once the coral larvae developed directional swimming at three days old, larvae were photographed under a dissection microscope with a scale bar (ToupView, ToupTek^TM^) to assess the average larval sizes for each treatment. All images were processed in the ImageJ Fiji software program [55], manually tracing the surface area of the individual larvae. Larvae were categorised as normal or severely deformed if they appeared lumpy and their swimming ability was compromised (Fig 1I).

### Relationship between larval and settler sizes

Settlement substrata were added to the experimental cultures on day five. The settlement substrata consisted of two pre-conditioned travertine limestone tiles per experimental culture (n = 120 tiles in total), with each tile measuring 5 x 5 x 1 cm. The travertine tiles were biologically conditioned by deployment on dead coral patches amongst live coral colonies at West Pelorus (−18.543908, 146.488480) ~2 months prior to coral spawning and lightly rinsed of sediment before use. Once the settlement tiles were added to the cultures, aeration was significantly reduced to allow larvae to attach and settle to the tiles. Settlement cultures were left for three days to allow larvae to firmly attach and metamorphose onto the substrata, carrying out daily 25% water changes. All tiles were then monitored under dissecting microscopes with cool LED lights, and all settlers were photographed with scale bars to determine their size.

### Settlement success of deformed larvae

A separate trial was conducted when larvae were five days old to determine whether normal-appearing larvae and deformed larvae settled at comparable rates when presented with a settlement cue. Settlement assays were performed in 6-well plates, with each well containing 10 mL of 1 µm UV-filtered seawater and a ~ 5 x 5 mm live chip of crustose coralline algae, *Porolithon onkodes* [53]. Five larvae from each group were individually collected using a pipette and added to separate wells. Negative controls consisted of wells with no settlement cues. Settlement assays were incubated in the temperature-controlled room alongside larval cultures and assessed after 24-hours under a dissection microscope. Larvae were categorised as: metamorphosed (settled spat), swimming, elongated and attached, elongated but not attached, and dead.

### Data analysis

The effect of collection methods on embryo condition, including both developmental stage and physical integrity, was tested with a Dirichlet regression model (package: DirichletReg, [56]) for each time point to determine differences in the composition of the different embryo categories. The compositional differences were then explored using General Linear Models (GLMs) with binomial error structure, followed by pairwise comparisons (package: emmeans, [57]), analysing proportional differences across the embryo categories.

Differences in larval diameter were modelled using a Linear Mixed-Effects Model (LMER) (package: lme4, [58]) with a Gaussian error structure to determine the effects of the collection method and timing of collection, as well as their interaction. Individual tanks were nested as a random factor in their treatments. To evaluate the significance of the fixed effects and their interactions, a Type III ANOVA was carried out (package: car, [59]), followed by pairwise comparisons to determine where the differences in average larval sizes were observed.

Settler diameter was analysed using a linear model with a Gaussian error structure to determine the effect of collection time and method. Due to the overall low settlement among replicates, their interaction could not be tested. The analysis was followed by pairwise comparisons to determine where the differences appeared. A linear regression model using a Gaussian error structure was used to determine if there was a relationship between average larval size and average settler diameter, with each data point representing the average larval and settler diameter for each treatment.

GLMs with quasi-binomial variance structure were used to determine the proportional difference in deformity rate across treatments, the relationship between deformity rate and larval size, and to compare settlement success between normal and deformed larvae. The quasi-binomial variance structure was chosen to account for both overdispersion and underdispersion observed in the datasets.

All model fits were evaluated through visualisation using residual and Q-Q plots to determine if model assumptions were met. Statistical analyses were carried out using the statistical program R [60].

## Results

### Effect of collection method and timing on embryo development

**Fig 2:**
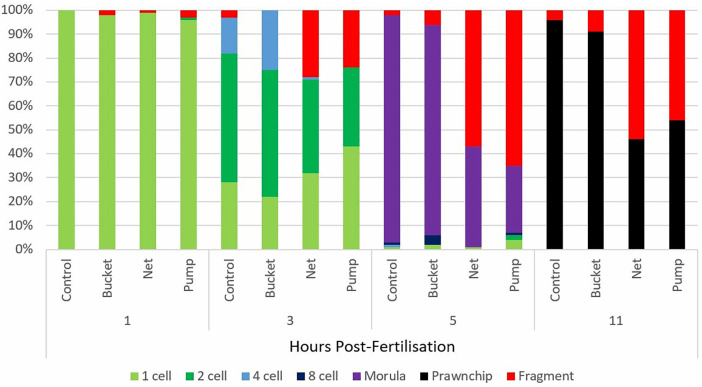
Average percentage of coral embryos in different developmental stages for the four time points and three collection treatments. Seven different development stages were classified; 1-cell (light green), 2-cell (dark green), 4-cell (blue), 8-cell stage (dark blue), morula (purple), prawn chip (black), and embryos that were classified as deformed and fragments (red). Treatments consisted of four time points: 1, 3, 5, and 11 hours post-fertilisation, and three collection methods: bucket, net, and pump, including control samples before each collection replicate (n = 5).

Collection methods significantly affected the integrity of developing embryos over time (Fig 2). At the first collection time point completed within the first hour post-fertilisation, none of the single-cell eggs or embryos had cleaved in the controls (i.e., in the fixed samples before collection method was applied). All collection methods had > 96% intact cells and embryos and showed no significant differences in the proportion of the single cell stage compared to controls, despite small numbers of fragmented eggs present.

Three hours post-fertilisation, most embryos had cleaved into their two- and four-cell stages, with >50% in the two-cell stage and 25% in the four-cell stage. The samples collected with the bucket had no significant effect on the proportions of embryos in different developmental stages compared to controls. In contrast, the net and pump collection methods significantly decreased the proportions of embryos in the two- and four-cell stages compared to the controls and bucket collection method (net; **p* *= 0.014 and **p* *= 0.018, pump; **p* *= 0.003 and **p* *= 0.003, respectively). Concurrently, relatively high levels of fragments and deformed embryos were observed for netted and pumped samples, with an average of 28% and 24%, respectively. However, the proportion of fragmented and deformed embryos in the netted samples was only marginally different to the controls (**p* *= 0.083).

Once embryos had reached the morula stage (defined as >8 cells) at five hours post-spawning, they were much more fragile, which was evident in the large percentage of fragments and deformed embryos in the netted and pumped samples (57–65%). In comparison, very low levels were observed in controls (<2%) and samples collected with the bucket (5%). The ratios of morulae and fragments and deformed embryos in the pumped and netted samples were significantly affected by the collection method (net; **p* *= 0.034 and **p* *< 0.001, pump; **p* *< 0.001 and **p* *< 0.001, respectively). The netted and pumped samples contained significantly fewer morulae than controls and samples collected with the bucket (**p* *< 0.001). Furthermore, netted samples had significantly more morula-staged embryos than pumped samples (**p* *= 0.005), which had the highest proportion of fragments and deformed embryos (Fig 2).

At 11-hours post-fertilisation, embryos had developed into their prawn chip stage and remained fragile and susceptible to fragmentation. The control and bucket samples had higher proportions of embryos in the prawn chip phase (91–96%) than the netted and pumped samples (46–54%). Correspondingly, the proportion of fragments and deformed embryos in pumped and netted samples was significantly higher (**p* *< 0.001) than in the control and bucket samples and showed similar patterns to those observed at five-hours post-spawning (Fig 2).

### Effect of collection method and timing on larval size

Larval size three days after fertilisation was significantly affected by the interaction between the method and timing of spawn and embryo collection (**p* *< 0.001).

#### Temporal changes in larval size within each collection method.

**Fig 3:**
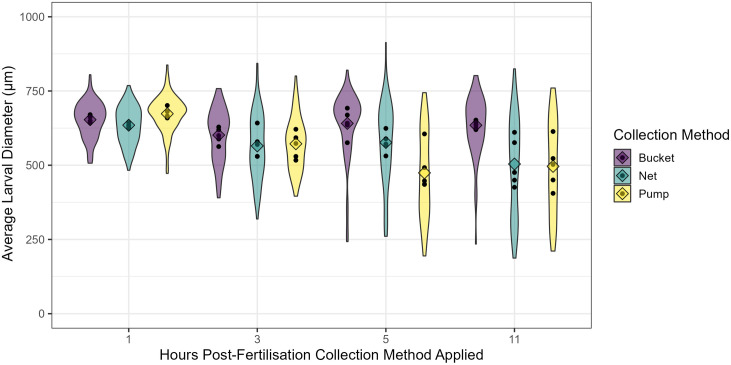
Average larval diameter at 3 days following spawning for each collection method and time point. Collection method treatments; bucket (purple), net (teal), and pump (yellow) at four different time points; 1, 3, 5, and 11 hours post fertilisation (t_0_, t_1_, t_2_, and t_3,_ respectively).. Diamonds indicate the average of the treatments, and black dots indicate the average of each treatment replicate (n = 5). Data were visualised as a violin plot to highlight the distribution of larval sizes in each treatment. Interaction between the method and timing of spawn and embryo collection (**p* *< 0.001).

At the first sampling point (t_0 _= one-hour post-fertilisation), larvae that developed from the different spawn collection techniques had similar sizes across treatments, with an average larval diameter of 654 ± 19 µm (Fig 3). Coral spawn collected by bucket consistently showed no effect on average larval size over time, with average larval diameter ranging from 601–653 µm. In contrast, larvae developed from spawn samples collected using the diaphragm pump and net resulted in pronounced fragmentation of some embryos as they increased in cell complexity and displayed a larger range in larval sizes. Netted samples collected at three- and five-hours post-fertilisation had slightly reduced average larval sizes (565 ± SE 11.8 µm and 577 ± SE 16.7 µm, respectively), although they were not significantly different from the first sampling point. Only the netted samples from 11-hours post-fertilisation (t_3_) resulted in statistically smaller larvae compared to the first sampling point (t_0_) (**p* *= 0.002), with larvae 21% smaller on average. All samples using the diaphragm pump had significantly smaller average larval sizes at all time points compared to the first sampling point (t_1_ **p* *= 0.025, t_2-3_ **p* *< 0.001), showing 15–30% reductions in average diameter.

#### Method-specific effects on larval size at discrete sampling times.

There were no significant differences in average larval sizes three days after fertilisation between the collection methods for the first two time points, one- and three-hours post-fertilisation (t_0,_ t_1_). However, by five-hours post-fertilisation (t_2_), pumping significantly reduced the average larval size compared to the samples collected by the bucket (**p* *< 0.001), but these were not significantly different from larvae developed from netted samples (**p* *= 0.067). At the final sampling point, 11-hours post-fertilisation (t_3_), both netted and pumped samples resulted in significantly smaller larvae, with 21–22% reductions in diameter compared to the samples collected by the bucket (**p* *= 0.002).

### Effect of larval size on settlers

**Fig 4:**
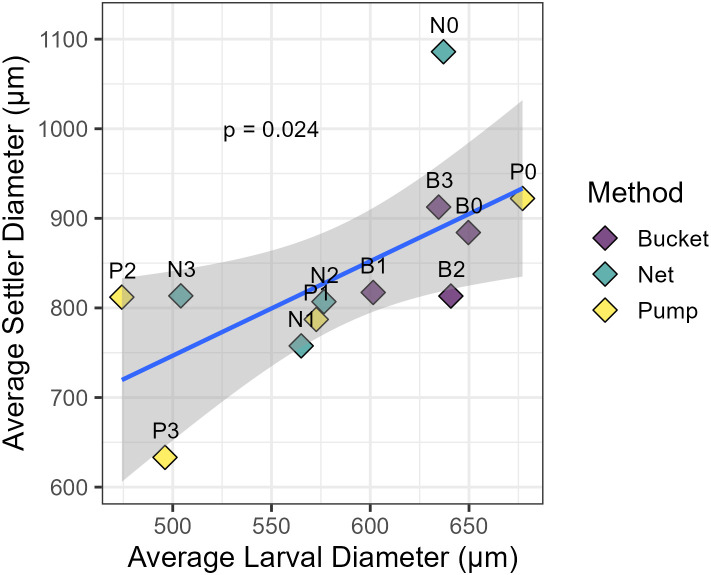
Correlation between average larval and settler diameter (µm) for each collection method and time point. Collection method treatments; bucket (purple), net (teal), and pump (yellow) at four different time points; 1, 3, 5, and 11 hours post fertilisation (t_0_, t_1_, t_2_, and t_3_ respectively). Tank ID refers to the collection method and time point (e.g., B0 = Bucket t_0_, t_0_ = one-hour post-fertilisation). Grey area indicates 95% confidence interval. Relationship between average larval diameter and settler diameter (**p* *= 0.024).

Linear regression showed a significant relationship between average larval diameter and settler diameter (**p* *= 0.024), which explained 41% of the variance (*R*^*2 *^= 0.414; Fig 4). The size of the newly settled spat was affected by both collection method and time of collection (**p* *< 0.001 and **p* *= 0.013, respectively) (*R*^*2 *^= 0.323). Settled spat from the first collection point, one-hour post-fertilisation, were significantly larger than all later time points (t_1_ **p* *< 0.001, t_2_ **p* *= 0.002, and t_3_ **p* *< 0.001), while all other time points showed no significant differences from each other. Samples collected by net resulted in significantly larger settlers compared to settlers from pumped larvae (**p* *= 0.012). Although the average diameter of settlers from larvae collected by bucket was consistently higher than the two other methods at time points t_1-3_ with an average diameter of 884–913 µm, compared to 807–845 µm for netted samples and 724–853 µm for pumped samples, variability in larval sizes from the net and pump were too high and replication too low to detect any significant difference.

### Larval deformities

**Fig 5:**
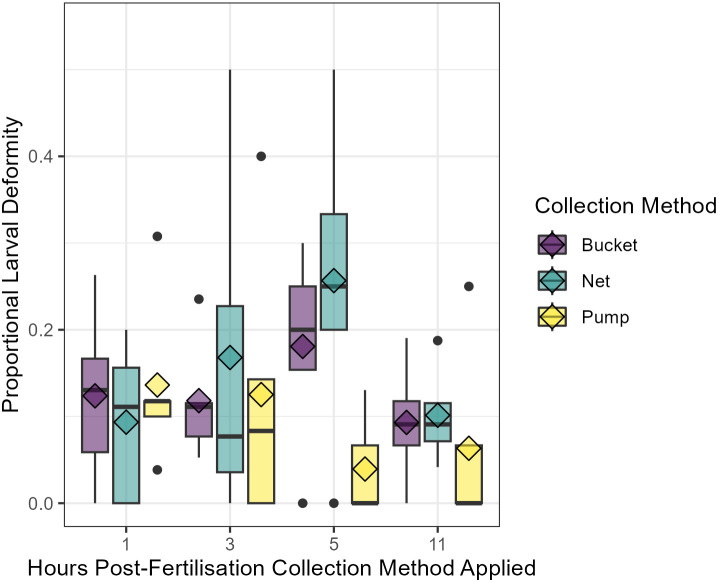
Proportion of deformed larvae at 3 days after fertilisation for each collection method at each sampling point. Collection methods; bucket (purple), net (teal), and pump (yellow) at each sampling point 1, 3, 5, and 11 hours post-fertilisation. Diamonds indicate the average of the treatments (n = 5). Data visualised with boxplots; horizontal black lines indicate the median value, and the coloured areas above and below the line indicate the upper and lower quartiles of the data, with the extended lines indicating maximum values, while the black dots are outliers.

There was no statistically significant difference in the proportion of deformed larvae between treatments three days post-fertilisation. However, the highest proportions of deformed larvae were observed five hours post-fertilisation (t_2_) in samples collected using the net (25.7%) and bucket (18.1%) methods. In contrast, samples collected using the pump at the same time point showed the lowest proportion of deformed larvae (3.9%) (Fig 5).

**Fig 6:**
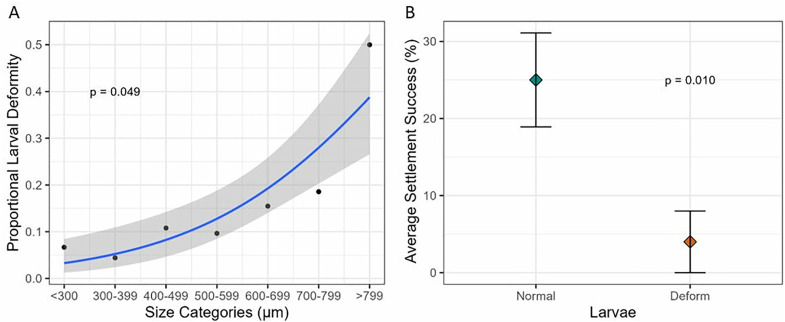
Proportional deformities for each size class and average settlement success. A) Proportion of deformed larvae in each size class of larvae grouped by average diameter (µm), with the blue line indicating the correlation between the data points and the grey area describing the 95% confidence interval. B) Average settlement success for normal (blue) and deformed (red) larvae in settlement assays, with coloured diamonds indicating the average settlement success and whiskers indicating standard error (±SE).

A significantly positive correlation was observed between the average diameter of larvae and the proportion of larvae that were deformed (**p* *= 0.049, *R*^*2 *^= 0.567), with smaller larvae potentially less prone to deformities (Fig 6A). Concurrently, five-day settlement assays revealed that only 4% of deformed larvae settled successfully when exposed to chips with live *P. onkodes*, while 25% of the normal larvae settled successfully (**p* *= 0.010) (Fig 6B).

## Discussion

Upscaling coral spawn collection is critical to increasing the production of coral larvae for reef restoration [2,11,19]. Access to predicted mass coral spawning times in many Indo-Pacific reef regions [61] combined with the ability to predict locations of spawn slicks [22] provide the foundation for large-scale harvesting of coral gametes [5,29]. With mass coral spawning events on the GBR limited to a few nights after two or three full moon periods each year [6,12], and coral embryos becoming increasingly fragile in the initial hours following spawning [23,33], determining how different collection methods affect the integrity of embryos collected at different time points and subsequent larval development is essential for the mass production of coral larvae. This study is the first to directly compare different methods of collecting coral spawn slick samples on the integrity of embryos and larval development for upscaling larval production.

From the results of our study, it is evident that embryos are significantly affected by different collection methods and the time of collection, with embryos showing increased levels of fragmentation as time progressed due to the lack of a protective wall around dividing cells [33,39]. Within the first hour after eggs and sperm were combined for fertilisation, before cell division had begun, the collection method had minimal impact on embryo integrity. Yet, when the developing embryos were at their two- and four-cell stages three hours post-fertilisation, the samples collected by bucket had an average of 15% of larvae in the four-cell stage, while pumped and netted samples had less than 1%. The data indicate that disruption to embryo development increased the number of fragments and deformed embryos in netted and pumped samples as well as the number of single cells, which were smaller, consistent with the results of previous studies [33,34]. As the embryos continued to develop and increase in complexity (5–11 hours post-fertilisation), collecting samples using the diaphragm pump and net had significant adverse effects on the integrity of the embryos, with more than 45% of the samples consisting of fragments and deformed embryos. The use of highly fragmented embryos in cultures increases the amount of dead and decaying cells, which reduces water quality and negatively affects developing coral larvae in conventional land-based aquaculture systems, rendering them suboptimal for larval cultures [2,9,23]. In comparison, collection by bucket had a negligible impact on the embryos throughout time, when embryos reach more delicate stages of development.

Results from this study indicate that the different collection methods exert varying levels of shear stress on the embryos. The collection method using a bucket is considered the least stressful for developing embryos, as they only move a short distance over the lip of the collection container and have limited contact with the sides of the container, explaining why this method has been widely used in gamete collection [10,23–25,27]. Nevertheless, it has limited scalability and requires dense spawn slicks. In contrast, collection using nets effectively concentrates dispersed coral spawn and has the potential for larger-scale collections [29]; however, the embryos likely experience higher levels of shear stress when they come in contact with the mesh nets as the water filters through the net, which results in increased fragmentation and smaller cell sizes. Industrial pumping of coral spawn slicks represents additional sources of shear stress in the form of flow acceleration and pressure changes [19,62]. However, the impact on embryo integrity was found to be similar to the collection method using nets in this study, and it is apparent that eggs and early-stage embryos can withstand relatively high levels of shear stress prior to cleavage.

Larvae that developed when the different spawn collection techniques were administered at one-hour post-fertilisation had similar sizes across treatments, with an average larval diameter of 654 ± 19 µm, consistent with other observations for *A. valida* larvae on the GBR [63]. While larvae from the samples collected using the bucket maintained large average larval sizes regardless of the collection time, a reduction in average larval size was observed for samples collected by net and pumping at the later time points. The average sizes of larvae from netted samples were generally larger than larvae from pumped samples until 11 hours post-spawning, when they developed into their prawn chip stage. These results are consistent with previous studies investigating coral embryo fragmentation and their ability to develop into smaller viable larvae [33,34,39]. Furthermore, the results from our study support the deduction that an observed larval survival rate of 120% when pumping trials occurred two hours post-fertilisation was an artefact of embryo fragmentation into smaller propagules that developed into smaller but healthy larvae [19]. Trade-offs between embryo size and fitness are important considerations, as smaller larvae have been found to have lower survival, likely due to reduced energy stores [39].

In agreement with earlier studies [33–35], the smaller larvae in our study were able to settle, and the size of the settlers was directly affected by larval size. The largest settlers were from collections that took place before embryo cleavage, while the smallest settlers were from pumped larvae at 11 hours post-spawning. This has important implications for post-settlement survival, which represents a significant bottleneck in coral restoration [2], as a reduction in size prolongs the time before reaching size-escape thresholds [43].

In this study, we observed that size did not always reflect the health of the larvae as we explored the occurrence of larval deformities and settlement abilities. Despite being alive and moving, deformed larvae were irregular in shape and unable to perform directional swimming and, importantly, had significantly reduced settlement success. Abnormal larvae such as those seen in the current study have been observed in previous studies when exposing coral larvae to pollutants [64,65].

In our study, the rate of larval deformities was the highest in samples collected at three- and five-hours post-spawning and most prevalent in netted samples (up to an average of 25%). A strong positive correlation between the rate of deformities and increasing size was observed. This result indicates that while larvae from samples collected by net at these time points were not significantly smaller than the larvae from the first time point, the health of some of these intact larvae was compromised. Thus, lower levels of shear force may have caused deformities (distortion of cells) rather than fragmentation into smaller embryos, observed as smaller larvae in pumped samples displaying higher levels of shear force [62]. These findings represent a potential trade-off between larval size, deformity, and settlement success. Several studies have reported that embryo fragmentation can produce smaller larvae without reducing settlement ability [33–35]. However, survival outcomes can be species-specific. In a recent study, it was found that fragmented and deformed *Acropora kenti* embryos had substantially lower survival compared to intact ones, whereas *Acropora spathulata* showed only moderate reductions [66]. This suggests that smaller larvae may be less problematic for larval culturing than deformed larvae, which, in this study, showed reduced settlement success. However, post-settlement trade-offs are likely, due to the positive relationship between size and post-settlement survival [43,44,50].

A limitation of this study is the lack of direct tracking of larval survival. It is plausible that smaller, deformed larvae experienced reduced survivorship compared to larger individuals. Although deformities may arise across the full range of larval sizes, their implications for viability remain uncertain. To better disentangle the effects of physical stress on developmental trajectories and survival, future studies should incorporate longitudinal tracking of larval fate across size classes.

The results of our study show that the timing of spawn collection is important. If the coral spawn is collected immediately after spawning, it can be collected using any method without compromising the quality of the larvae. Pumping larval slicks has the potential to reduce the time required for collection and upscale collection, but does have a smaller window of opportunity compared to the other collection methods. The success of a previous pumping trial [19] is thought to be attributed to the timing during which it took place (two hours post-fertilisation), as our study showed higher levels of one-cell stage embryos and relatively low levels of unrecognisable fragments at three hours post-fertilisation, which increased significantly after this time point. Thus, vessels should arrive at predicted spawning sites before peak release to harvest and stock embryos in situ to effectively collect coral spawn. However, locating coral spawn slicks can be time-consuming, and additional time is needed for mobilisation. This, along with transport and stocking, introduces handling stress to the delicate embryos, potentially extending the process to the 3-hour timeframe trialled in the study, which may lead to fragmentation into smaller embryos. Additionally, culturing in situ exposes developing embryos to environmental conditions, such as wave action, that could result in further fragmentation. However, the improvement to in situ rearing pools developed over several years of trials has reduced wave impacts on cultures when exposed to adverse weather conditions [11,25,29].

Embryo fragmentation or polyembryony may be inevitable when collecting from wild coral spawn slicks, as the spawning times of different species vary [61], resulting in embryos in spawn slicks being at various stages of embryogenesis. With some studies suggesting polyembryony is a natural adaptation and more common in the wild than previously thought [33,36], a degree of fragmentation in wild cultures may be acceptable. However, coral spawn slick harvesting should aim to minimise shear stress that results in the fragmentation and deformation of embryos during the process. Based on our results, active collection methods using nets and pumps should be limited to the first hours following spawning, while collection by buckets could be utilised for an extended period after spawning and the following morning if the slick samples are dense and can be effectively collected. Passive collection methods, such as large-scale spawn catchers [11,25,29] that add no shear stress to the developing embryos, may also provide a viable option for larger-scale collections over extended time periods.

The outcomes from this study show how large-scale spawn collections can be done without compromising larval quality by careful consideration of the collection method and stage of embryogenesis that is collected following major spawning events. This is essential for overcoming bottlenecks in coral reef restoration planning, where maximising larval production and health while minimising handling stress is key to scaling up restoration efforts at ecologically relevant scales. While the Philippines and Great Barrier Reef have been primary regions for developing larval restoration techniques in recent years, coral spawning is well documented in many other regions around the world [5,16,61], and the potential applicability of these methods is expanding as knowledge and capacity grow in other reef regions.
